# Stroboscopic visual training in sport: a structured narrative review of effects, applications, and potential neurophysiological mechanisms

**DOI:** 10.7717/peerj.21740

**Published:** 2026-09-29

**Authors:** Qi Li, Zhijie Yan, Ruifeng Liu, Junchen Wu, Hu Yang

**Affiliations:** 1 School of Physical Education, Central China Normal University, Wuhan, China; 2 Sports Coaching College, Beijing Sport University, Beijing, China

**Keywords:** Stroboscopic visual training, Cognitive function, Motor performance, Neuroplasticity

## Abstract

Stroboscopic Visual Training (SVT) is an emerging intervention that periodically occludes visual input and creates a temporally constrained visual environment during perceptual or sport-specific tasks. This structured narrative review synthesizes current evidence on the effects of SVT on perceptual-cognitive function and sport performance and discusses its potential neurophysiological mechanisms. The available evidence suggests that, under restricted visual-input conditions, SVT may facilitate more efficient use of task-relevant visual information and may support anticipatory decision-making, visual short-term memory, and perception–action coupling. These effects appear more likely to occur in tasks requiring rapid reactions, precise judgments, and the processing of high-speed dynamic information, although the magnitude and transferability of these effects remain task-specific and inconsistent across studies. Preliminary neurophysiological evidence indicates that SVT has been associated with changes in event-related potential components, including N2 latency in motion-sensitive visual areas and P100 latency in the primary visual cortex. However, these findings should be interpreted cautiously because the current mechanistic evidence is limited, often based on small samples, and relies mainly on indirect neurophysiological measures. Although SVT is a controllable training approach that can be embedded within sport-specific practice, its ecological transfer to real competitive performance and its long-term effectiveness remain insufficiently established. Future research should focus on establishing dose–response models for SVT parameters, applying multimodal neurophysiological methods, improving methodological quality through active control conditions and follow-up assessments, and systematically examining task transfer and long-term retention to support more precise and evidence-informed applications of SVT in sport.

## Introduction

The critical role of visual function in athletic performance has been widely recognized (Lochhead et al., 2024; Guo et al., 2024; Buscemi et al., 2024). Visual and perceptual-cognitive functions are important contributors to performance in many sport contexts, particularly in open-skill and externally paced sports in which athletes must rapidly detect, interpret, and respond to dynamic environmental information. For example, in racket sports such as badminton and tennis, athletes are required to anticipate ball trajectory and opponent movement (Costa et al., 2023; Phomsoupha & Laffaye, 2015; Chen, Zulnaidi & Syed Ali, 2023), whereas in combat sports such as boxing and sanda, rapid cue detection, response selection, and action execution under time pressure are essential (Wu et al., 2024; Chen et al., 2017). Therefore, efficient visual information processing should not be regarded as a universal prerequisite for all forms of high-level athletic performance, but rather as a key factor that supports skilled performance in tasks requiring rapid perception–action coupling. Accordingly, sports vision training has gradually become an important component of modern athletic preparation, as it aims to enhance perceptual processing, attentional allocation, anticipation, decision-making efficiency, and action execution under competitive conditions.

Among the various approaches to sports vision training, stroboscopic visual training (SVT) has attracted increasing attention because it dynamically regulates visual input through periodic occlusion (Talwar, Smith & Lamberth, 2024; Jothi, Dhakshinamoorthy & Kothandaraman, 2025a; Fuerst, Lynch & Kohmescher, 2024; Das et al., 2023; Wu et al., 2025). The origins of SVT can be traced back to early intermittent-vision research, and its systematic empirical development accelerated in the early twenty-first century with the emergence of commercial stroboscopic eyewear (Fuerst, Lynch & Kohmescher, 2024). To date, several companies, including Vima and Senaptec, have introduced different models of such devices, typically operating at occlusion frequencies ranging from 5 to 30 Hz and widely applied in sport-specific training across multiple disciplines (Haberstroh, 2017). These systems use adjustable liquid crystal display (LCD) lenses to intermittently block visual information, forcing athletes to extract and process task-relevant cues within brief “visual windows” and thereby challenging the visual system in a controlled manner.

Current sports vision training can generally be divided into two broad paradigms: component training and naturalistic training (Appelbaum & Erickson, 2018). Component training targets basic visual functions through modular drills and can improve performance on specific tasks under controlled laboratory conditions (Deveau et al., 2015). However, because of its relatively low ecological validity, its benefits often transfer poorly to complex competitive environments (Appelbaum & Erickson, 2018). Naturalistic training, by contrast, embeds visual demands within sport-specific actions and situational contexts, offering greater ecological relevance and transfer potential. SVT appears to integrate key features of both paradigms. In practice, it is often embedded within sport-specific movements, thereby preserving elements of performance context. At the same time, intermittent visual occlusion introduces a controlled perturbation to the visual channel and increases visual-cognitive demands (Fig. 1). Rather than being directly comparable to physiological or mechanical overload strategies such as altitude training or resisted running, SVT may be more appropriately viewed as a constraint-based perceptual training approach. While these traditional methods rely on restricted input to induce compensatory adaptation (Carrolla et al., 2021; Wilkins & Appelbaum, 2020), SVT operates by intermittently reducing visual availability, thereby creating a controlled visual perturbation. This approach may encourage athletes to rely more efficiently on task-relevant cues and may strengthen perception–action coupling under sport-specific task demands. Owing to its relative safety, practical feasibility, and high repeatability, SVT is increasingly being regarded as a promising and scientifically grounded approach to cognitive intervention in sport (Das et al., 2023).

**Figure 1:**
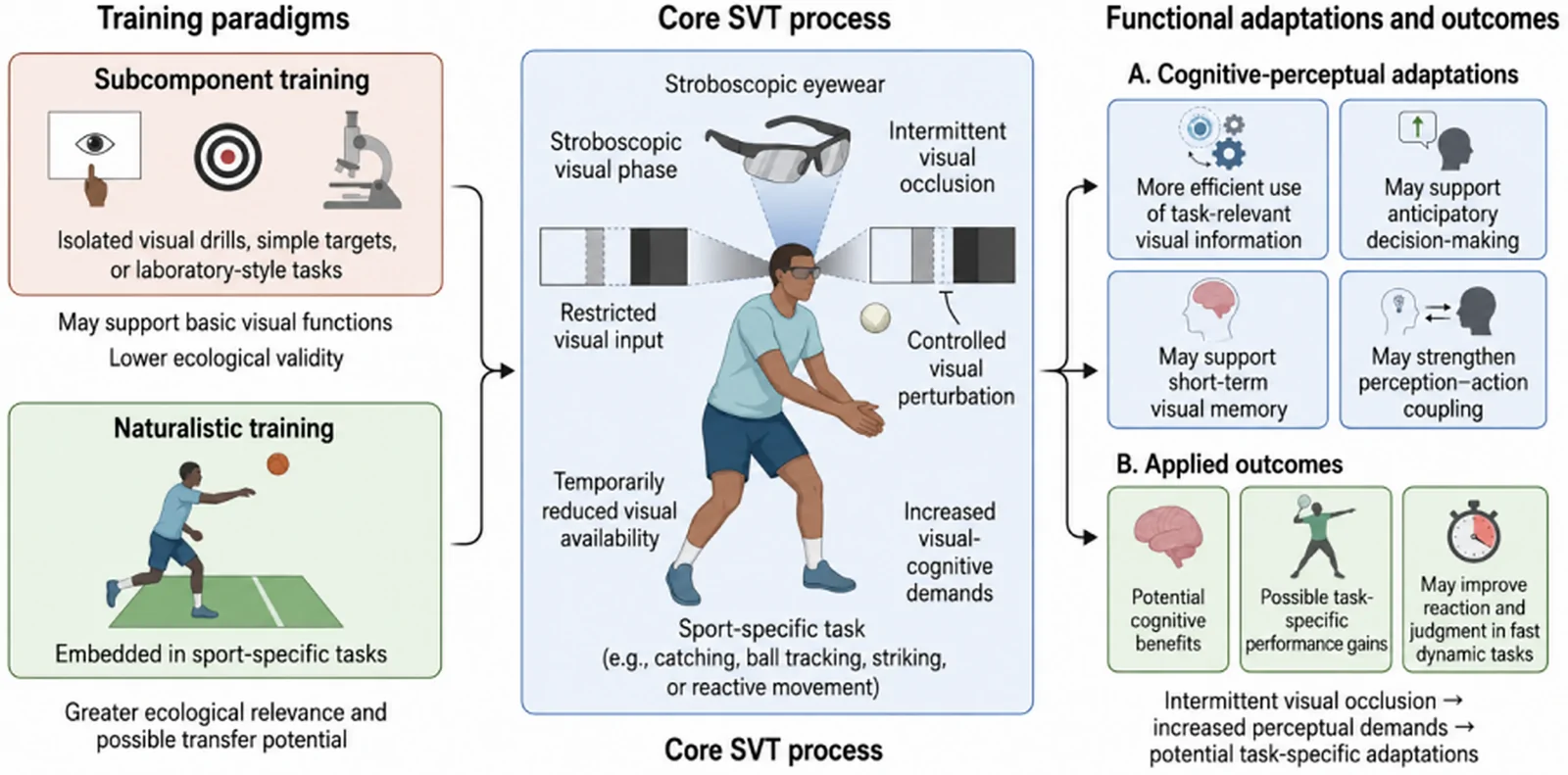
Conceptual framework of stroboscopic visual training in sport-specific practice. SVT, stroboscopic visual training. The figure shows how SVT may integrate component-based and naturalistic training by embedding intermittent visual occlusion in sport-specific tasks. This perturbation may increase visual-cognitive demands and reliance on task-relevant cues. Potential effects include changes in visual processing, anticipatory decision-making, short-term visual memory, and perception–action coupling, depending on task characteristics, training parameters, and individual differences.

In this review, related terms are used with distinct meanings. “Visual processing” refers to the early detection, encoding, and transmission of visual information, including visual acuity, motion perception, and early cortical responses. “Perceptual-cognitive function” involves higher-order processes such as attention, anticipation, decision-making, and visual short-term memory. “Visuomotor integration” describes the transformation of visual information into motor responses, while “perception–action coupling” refers to the link between information pickup and action execution in sport contexts. “Neural efficiency” is used cautiously as an interpretative concept, indicating that similar or improved performance may occur with reduced or more selective neural activation, and is not treated as direct evidence of neural adaptation without appropriate neurophysiological support.

Recent systematic reviews and meta-analyses have summarized the effects of SVT on physical performance, reaction time, movement accuracy, decision-making, cognitive function, and sport-specific performance (Dingirdan Gultekinler et al., 2025; Luo et al., 2025; Wang et al., 2025a; Guo et al., 2025; Ren et al., 2025; Vera et al., 2025; Wang et al., 2025b). However, these reviews mainly evaluated intervention effectiveness or pooled performance outcomes, whereas less attention has been given to a sport-specific conceptual synthesis linking perceptual-cognitive effects, sport-performance transfer, practical applications, and neurophysiological evidence. Therefore, this structured narrative review integrates current evidence on SVT across cognitive, sport-performance, and neurophysiological domains. It examines SVT’s effects on perceptual-cognitive function and sport-performance transfer, the supporting neurophysiological evidence, and key methodological limitations in competitive sport.

## Methods

### Review design

This article was conducted as a structured narrative review designed to provide a comprehensive and thematically organized synthesis of the existing literature on SVT. This approach was selected because the available SVT literature is heterogeneous in terms of participant characteristics, sport contexts, intervention protocols, stroboscopic settings, outcome measures, and neurophysiological assessment methods. Therefore, the purpose of this review was not to conduct a formal systematic review or meta-analysis, but to critically integrate current evidence across cognitive, sport-performance, and neurophysiological domains.

### Search strategy

Electronic searches were conducted in PubMed, Web of Science, SPORTDiscus, and Google Scholar from database inception to 17 January 2026. The search terms primarily included “stroboscopic visual training,” “stroboscopic vision,” “strobe eyewear,” “stroboscopic eyewear,” “intermittent visual occlusion,” and “visual occlusion training.” These terms were combined, when appropriate, with sport- and performance-related terms such as “sport,” “athlete,” “performance,” “reaction time,” “anticipation,” “decision-making,” “visual cognition,” “perceptual-cognitive function,” “eye–hand coordination,” “EEG,” “ERP,” and “fMRI.” Google Scholar was used as a supplementary source rather than as a primary systematic database. Targeted searches were conducted using key SVT-related terms, and relevant results were screened; records lacking sufficient information or relevance were not included in the main synthesis.

A representative Boolean search string was as follows: (“stroboscopic visual training” OR “stroboscopic vision” OR “strobe eyewear” OR “stroboscopic eyewear” OR “intermittent visual occlusion” OR “visual occlusion training”) AND (“sport” OR “athlete” OR “performance” OR “reaction time” OR “anticipation” OR “decision-making” OR “visual cognition” OR “perceptual-cognitive” OR “eye–hand coordination” OR “neurophysiological” OR “EEG” OR “ERP” OR “fMRI”). The syntax was adapted according to the requirements of each database. Google Scholar was used as a supplementary source through targeted searches and citation tracking rather than as a primary systematic database. In addition, backward reference checking and forward citation tracking were performed for key original studies and relevant review articles to identify additional literature.

### Study relevance and selection

Studies were considered relevant if they examined SVT or intermittent visual occlusion in relation to cognitive function, perceptual-cognitive performance, sport-specific performance, motor behavior, or neurophysiological mechanisms. Studies were not included in the main synthesis if they were unrelated to SVT, lacked a sport- or performance-related context, were not available with sufficient full-text detail, or provided insufficient methodological or outcome information for interpretation. Because this article was designed as a structured narrative review rather than a PRISMA-based systematic review, no formal independent dual-screening procedure or record-level flow diagram was performed. Instead, study selection was based on topical relevance, methodological clarity, and contribution to the main thematic domains of the review. No formal language restriction was applied; however, studies required sufficient full-text information for interpretation. The studies included in the synthesis are listed in Tables 1, 2 and Tables S1, S2.

**Table 1:** Effect of stroboscopic visual training on cognition.

Author/year	Participant characteristics	Stroboscopic device	Strobe settings	Training frequency/duration	Total stroboscopic exposure	Testing tasks	Key findings
Zwierko et al. (2024a)	50 youth volleyball players (16.5 ± 0.6 years)	Senaptec Strobe glasses	15 Hz → 9 Hz (duty cycle 50% → 70%), progressively adjusted weekly	6 weeks, 3 sessions per week	~270 min	Laboratory: simple/complex reaction time, saccadic tracking; Field: reactive agility (5-cone shuttle run)	Simple motor time ↓, complex reaction time ↑, saccadic dynamics ↑, reactive agility ↑
Zwierko et al. (2024b)	50 youth volleyball players (16.5 ± 0.6 years)	Senaptec Strobe glasses	15 Hz → 9 Hz (duty cycle 50% → 70%), progressively adjusted weekly	6 weeks, 3 sessions per week	~270 min	Laboratory: saccade acceleration; Field: CODS/RA tests → REAC-INDEX	REAC-INDEX ↑
Vasile & Stănescu (2024)	17 elite youth climbers	Not specified	Not reported	1 session per week × 20 sessions (1-year period)	Not reported	Cognitive tests (spatial skills/reaction), climbing-specific tests	On-sight/red-point ↑ Spatial visual processing speed ↑ Cognitive agility ↑
Zwierko et al. (2023)	22 elite male handball players	Senaptec Strobe glasses	Frequency adjusted over time (not specified)	3 sessions per week × 6 weeks + 4-week retention period	Not reported	Visual evoked potentials (P100 latency/amplitude)	P100 latency ↓ Visual conduction efficiency ↑
Fortes et al. (2023)	28 male professional soccer players (18–32 years)	Nike Vapor Strobe^®^	100 ms visible/210 ms opaque (~3.23 Hz)	8 weeks (3 sessions per week)	Not reported	Multiple object tracking (MOT), anticipation tasks, decision-making tasks	Anticipation skills ↑ Perceptual–cognitive skills →
Hülsdünker, Fontaine & Mierau (2023)	26 college students (non-athletes)	Senaptec Strobe glasses	5 Hz (40% duty cycle)	Single-session experimental condition (non-longitudinal)	Not reported	Visuomotor response task (EEG recording of N2 component)	Reaction time under stroboscopic conditions ↓, N2 latency ↑, N2 amplitude ↓, central visuomotor processing speed ↓
Hülsdünker, Gunasekara & Mierau (2021a)	32 youth badminton players	Senaptec Strobe glasses	15 Hz → 8 Hz, dynamically adjusted	10 weeks, 45 min per week (average)	450 min	Laboratory: VMRT; Field: catching accuracy, net-play performance	VMRT ↓Field-measured reaction speed ↑
Ellison et al. (2020)	62 male physically active participants	Nike Vapor Strobe^®^	100 ms visible/150 ms opaque	Acute training: 4 sessions, each with 6 sets × 20 strobe responses (total 480), 7–8 min per session	~32 min (4 × 8 min)	1. Sport Vision Trainer™ – eye–hand coordination (EHC) 2. e-Prime visual search (VS) task	SG group: significant improvement ↑ in EHC task at immediate, 10-min, and 10-day retention tests; no significant change → in VS task
Ballester et al. (2017)	20 male college students (mean age: 23.15 years)	Nike Vapor Strobe^®^	100 ms visible/150 ms opaque (4 Hz)	Single-session experiment: 4 sets × 30 trials, 10–11 min	10–11 min	Coincidence-anticipation tasks, psychomotor vigilance tasks	Constant error (CE), variable error (VE), reaction time/variability in vigilance tasks →
Appelbaum et al. (2016)	25 collegiate softball players (15 in the intervention group, 10 in the control group)	Nike Vapor Strobe^®^	Initial level 3 (100 ms transparent/150 ms opaque)	2 sessions per week × 11 weeks, 5–10 min per session (average 11.4 sessions)	~165 min (11 weeks × 2 sessions/week × 7.5 min/session)	Nike Sensory Station–9 tasks: near–far quickness, target capture, Go/No-Go, *etc*.	Intervention group: improvements ↑ in near–far quickness, target capture, and Go/No-Go tasks
Wilkins & Gray (2015)	30 athletes (12 females, 18 males; mean age: 22.5 years; training experience: 5–20 years)	PLATO visual occlusion glasses	VSR group: Level 1–8 CSR group: fixed at Level 1	10 sessions over 6 weeks, 20 min per session	Total duration ~200 min	One-handed catching, useful field of view (UFOV), motion-in-depth sensitivity (MIDS) tests	Between-group: no change → Significant correlations between improvements in UFOV single-task, MIDS, and catching performance
Holliday (2013)	16 general participants (allocated to experimental and control groups)	Nike Vapor Strobe^®^	Not reported	9-day training (with immediate, 2-week, and 4-week tests)	Not reported	Dynamic visual acuity test (NeuroCom system), catching performance	Dynamic visual acuity (left/vertical/up–down directions) ↑ Catching success rate ↑
Smith & Mitroff (2012)	30 university community members (20–29 years; mixed sex)	Nike Vapor Strobe^®^	100 ms visible/150 ms opaque (4 Hz)	Five training sets (10 trials per set), single session 5–7 min	5–7 min	Bassin anticipation timer	Absolute error, early/late response bias, variable error ↓
Appelbaum et al. (2012)	84 participants (Duke University community members and varsity athletes: men’s soccer, women’s soccer, men’s basketball)	Nike Vapor Strobe™ glasses	Level 1–6 (100 ms transparent, 67–900 ms opaque)	Two training sessions (27 min each); team training: 6–7 sessions (soccer), 5–6 sessions (basketball), 15–45 min per session	Laboratory: 54 min; Team: total duration variable	Partial report memory task (presented letter arrays, delayed recall)	Short-term memory retention ↑ Training effects maintained for 24 h
Appelbaum et al. (2011)	157 participants (laboratory group, varsity football players, and ultimate frisbee club members)	Nike Vapor Strobe™ glasses	Level 1–6 (100 ms transparent, 67–900 ms opaque)	Laboratory: 2–4 sessions (27 min each); Team: rugby 9–10 sessions (15–30 min each), ultimate frisbee four sessions	Laboratory: 54–108 min; Team: total duration variable	Motor consistency threshold (central/peripheral), UFOV dual-task, multiple object tracking (MOT)	Central motor sensitivity and transient attention (UFOV dual-task) ↑; peripheral motor sensitivity and sustained attention →

**Notes:**

↑ indicates improvement; ↓ indicates decline; → indicates no change.

CODS, change-of-direction speed; RA, reactive agility; REAC-INDEX (s), RA–CODS visuomotor response index; on-sight/red-point, successful route completion on the first attempt without prior exposure or route information/successful completion after repeated practice without falls and without additional protection; MOT, multiple object tracking; EHC, eye–hand coordination; UFOV, useful field of view; MIDS, motion-in-depth sensitivity.

**Table 2:** Effect of stroboscopic visual training on exercise performance.

Author/year	Participant characteristics	Stroboscopic device	Strobe settings	Training frequency/duration	Total stroboscopic exposure	Testing tasks	Key findings
Zwierko et al. (2024b)	50 youth volleyball players	Senaptec Strobe glasses	Fixed frequency: 2.0 Hz	6 weeks (2 sessions/week × 30 min)	360 min	Blocking reaction speed test	Experimental group: reaction speed ↑, but effects dissipated after 4 weeks →
Elhachemi & Issam (2024)	U13 soccer goalkeepers	Senaptec Strobe glasses	Fixed frequency: 0.2 Hz	7 days (2 sessions/day × 25 min)	350 min	Reaction speed/ball control test	Reaction speed ↑, ball control accuracy ↑
Zwierko et al. (2024c)	22 male soccer players	Senaptec Strobe glasses	Fixed frequency: 0.5 Hz	Single-session warm-up (20 min)	20 min	Reactive agility test	Dribbling agility ↑, performance under fatigue ↓
Liu et al. (2020)	24 NCAA Division I collegiate baseball batters	Nike Vapor Strobe™ glasses	Dynamic mode: 0.3–5 Hz, random switching	3 sessions/week × 30 min, total ~8.5 h	510 min	Hitting practice performance	Hitting angle ↑, hitting distance ↑
Kroll et al. (2023)	13 female volleyball players	VisualEyes Strobe glasses	Dual-mode comparison: 1.75 Hz *vs*. 4 Hz	Single-session test	30 min	Jump landing GRF analysis	Reaction index ↓ at 1.75 Hz, ground contact time ↑
Palmer, Coutts & Fransen (2022)	Youth soccer players	EyeTech Strobe system	Fixed frequency: 0.7 Hz	4 weeks (3 sessions/week × 20 min)	240 min	Ugent dribbling test	Dribbling time →, number of ball contacts →
Beavan et al. (2021)	84 amateur male soccer players	NeuroTracker Strobe glasses	Fixed frequency: 0.5 Hz	2 training sessions (90 min each)	180 min	Footbonaut passing test	High-level group: passing accuracy ↓, time ↑; Low-level group: accuracy →
Edgerton (2018)	12 softball players	Stroboscopic Pro glasses	Fixed frequency: 4.0 Hz	4 weeks (3 sessions/week × 25 min)	300 min	Ground ball reception test	Experimental group: catching accuracy ↑ Expert group: greater improvements
Wilkins, Nelson & Tweddle (2018)	Six elite youth soccer goalkeepers	Senaptec Strobe glasses	Fixed frequency: 1.2 Hz	7 weeks (5 sessions/week × 10 min)	350 min	10-item visual–cognitive test battery	Visual reaction time ↑, other measures →
Schootemeijer & Visch (2017)	13 youth tennis players	Vision Coach Pro system	Random mode: 0.5–3.0 Hz, irregular variation	3 weeks (2 sessions/week × 20 min)	120 min	Forehand/backhand hitting accuracy test	Hitting accuracy ↑
Fransen et al. (2017)	189 youth soccer players	Strobe Specs Glasses	Dual-condition experiment: 0.3 Hz (low)/1.0 Hz (high)	Single-session test (15 min each)	30 min	Dribbling speed test	High-frequency mode: high-speed dribblers’ performance ↓
Mitroff et al. (2013)	Six NHL ice hockey players	SPARQ Vapor Strobe glasses	Progressive: starting at 2.0 Hz, +0.5 Hz per week	7 weeks (3 sessions/week × 20 min)	420 min	On-ice shooting/passing test	Experimental group performance ↑, control group →
Clark et al. (2012)	Collegiate baseball team	Vision UP Stroboscopic system	Fixed frequency: 3.0 Hz	6 weeks (4 sessions/week × 15 min)	360 min	NCAA game statistics	Batting average ↑, slugging percentage ↑

**Note:**

↑ indicates increase; ↓ indicates decrease; → indicates no change.

### Structured methodological appraisal approach

To improve the transparency of evidence interpretation, the methodological characteristics of the included studies were appraised using a structured approach informed by the principles of the Mixed Methods Appraisal Tool (MMAT). Because this article was designed as a structured narrative review rather than a formal systematic review, the appraisal was not used to exclude studies, generate numerical quality scores, or conduct quantitative risk-of-bias weighting. Instead, it was used to guide the narrative interpretation of evidence strength.

The appraisal considered the following features: clarity of study design, participant characteristics, sample size, presence and type of control condition, reporting completeness of SVT parameters, ecological validity of outcome measures, intervention duration, follow-up testing, and directness of neurophysiological evidence. Studies with controlled designs, clearly reported SVT parameters, sport-specific outcome measures, and follow-up assessments were interpreted as providing stronger evidence than uncontrolled, acute, case-based, multi-component, or laboratory-only studies. Mechanistic findings based on small samples, indirect outcomes, or a limited number of research groups were interpreted cautiously.

The methodological characteristics and appraisal considerations of the included studies are summarized in Tables S1 and S2.

### Narrative synthesis

Information was extracted narratively from the included studies, including participant characteristics, sport context, stroboscopic device, training parameters, outcome measures, main findings, and reported limitations. The evidence was synthesized thematically across three domains: cognitive and perceptual effects, sport-performance effects, and potential neurophysiological mechanisms. Findings from controlled intervention studies, sport-specific tasks, and studies with follow-up assessments were interpreted more strongly than findings from uncontrolled, single-session, or case-based studies. Mechanistic claims based on small samples, indirect outcomes, or a limited number of research groups were interpreted cautiously.

This review was not prospectively registered in PROSPERO because it was designed as a structured narrative review rather than a formal systematic review or meta-analysis. It was not intended to be a PRISMA-based systematic review; however, key elements of transparent reporting, including search sources, search terms, relevance criteria, methodological appraisal considerations, and narrative synthesis procedures, were described to improve reproducibility and interpretative transparency.

## Effects of svt on cognitive function

The effects of SVT on cognitive function can be organized into three interrelated domains: early visual processing, perceptual-cognitive function, and visuomotor integration or perception–action coupling. The following sections synthesize current evidence from the perspectives of visual information processing, anticipation and decision-making, visual memory and attention, and sport-related perception–action coupling (see Table 1 for details).

### Perceptual level: optimization of visual information processing efficiency

Basic visual processing represents a fundamental stage of early neural signal transmission and serves as an essential basis for higher-order cognition and motor performance. By intermittently restricting visual input and creating a more challenging visual environment, SVT may encourage athletes to use limited visual information more efficiently, thereby improving early perceptual processing and the transmission efficiency of the visual pathway.

Using visual evoked potentials (VEPs), Zwierko et al. (2023) examined the effects of a 6-week SVT intervention in handball players and found that P100 latency in the peripheral visual field of the dominant eye was shortened by 9.3 ms in the experimental group, suggesting that SVT can significantly enhance neural conduction speed within the visual system. In a short-term intervention using the Nike Vapor Strobe^®^ system in soccer players, Holliday reported a 12% improvement in dynamic visual acuity (DVA) for horizontally moving targets, with additional gains observed in the upward and leftward directions. However, follow-up assessments showed that these benefits were no longer significant at 24 h and 2 weeks after the intervention, indicating that the neural modulation induced by short-term SVT may be transient (Holliday, 2013). In addition, Hülsdünker, Fontaine & Mierau (2023), using computerized reaction-time tasks combined with electroencephalography, found that 5 Hz stroboscopic stimulation significantly delayed the N2 component in the motion-sensitive cortical area and reduced N2 amplitude, whereas no such changes were observed under the screen-shutter condition. This finding suggests that specific stroboscopic frequencies may modulate temporal coding in the visuomotor cortex and thereby influence early perceptual processing (Hülsdünker, Fontaine & Mierau, 2023).

Overall, current evidence suggests that SVT may improve basic visual processing, particularly in terms of neural conduction speed, dynamic visual function, and visuomotor information integration. However, whether these effects can be maintained over time and whether they generalize consistently across different perceptual measures remain unclear.

### Decision-making level: enhancement of anticipatory judgment and spatiotemporal integration abilities

#### Anticipation timing and judgment ability

Anticipation refers to the ability to predict potential events before all relevant information has been fully presented, based on prior knowledge and contextual cues (Sun, Wang & Xia, 2014). Anticipatory timing, a core component of complex motor decision-making, reflects an athlete’s capacity to predict the temporal occurrence of events. By restricting visual input and simulating incomplete-information conditions, SVT may strengthen predictive judgment processes.

Early evidence supports this view. Smith & Mitroff (2012) used Nike stroboscopic eyewear in a Bassin anticipation timing task and found that a brief 5–7 min intervention significantly improved predictive accuracy, with absolute error reduced by 23% and the coefficient of variation of error reduced by 18%. The effect remained evident 10 min after the intervention, but the between-group difference disappeared after 10 days, suggesting that the adaptation induced by short-term SVT may be difficult to maintain over time (Smith & Mitroff, 2012). Ballester et al. (2017) extended this paradigm by applying 4 Hz stroboscopic stimulation in 20 university students and combining an anticipatory timing task with a psychomotor vigilance task. Their results showed stable constant and variable errors in the experimental group, further supporting the role of SVT in enhancing predictive ability (Ballester et al., 2017).

Importantly, the anticipatory benefits of SVT appear to be task-specific. Fortes et al. (2023) conducted an 8-week intervention in 28 male soccer players and found that both the stroboscopic and control groups improved in multiple object tracking (MOT) and decision-making performance, with no significant between-group differences. In contrast, both groups improved in anticipatory ability, with the stroboscopic group showing greater gains than the control group. These findings suggest that repeated SVT may be more effective for enhancing sport-specific anticipatory skills than for improving general perceptual-cognitive performance (Fortes et al., 2023).

Overall, current evidence indicates that SVT has the potential to improve anticipatory timing and judgment, particularly in sport-specific contexts. However, its long-term durability and transferability across tasks remain to be established.

#### Visual memory and attentional regulation

Visual memory is a key cognitive component of motor performance, involving the encoding, storage, and retrieval of visual information. Improved visual memory may help athletes rapidly identify environmental features and retrieve relevant experience (Liu & He, 2017). Existing studies suggest that the facilitating effects of SVT on visual memory are mainly reflected in improved short-term retention capacity and durability. Appelbaum et al. (2012) conducted a ball-catching training experiment in which the experimental group received progressive stroboscopic intervention. Participants showed significantly greater letter retention in a modified partial-report task, particularly under long-delay conditions, and the improvement persisted for more than 24 h, indicating that SVT may strengthen the encoding and consolidation of visual information (Appelbaum et al., 2012).

With respect to attention, the effects of SVT appear to be more evident for transient central attention than for sustained attention. Appelbaum et al. (2011) found that SVT significantly improved the accuracy of central tasks, whereas no significant improvement was observed in MOT performance, suggesting that its effects on complex sustained attention may be limited. Similarly, Wilkins & Gray (2015) reported in a 6-week ball-catching training study that the experimental group showed greater improvement than the control group in single-task accuracy on the useful field of view test, although the between-group difference did not reach statistical significance. This finding suggests that the observed gains may be related to restricted visual input rather than being uniquely attributable to the stroboscopic mode itself (Wilkins & Gray, 2015).

SVT may also facilitate divided attention. Argilés et al. (2025) compared stroboscopic eyewear training, action video game training, and a control condition in 30 sedentary adults. Although 1 h of stroboscopic training did not produce immediate improvements, MOT performance improved significantly at the 1-month follow-up, whereas no significant changes were observed in dynamic visual acuity (Argilés et al., 2025). These findings suggest that SVT may have greater effects on visual attentional tracking than on dynamic visual function, and that some of its benefits may emerge only after a delay.

Taken together, current evidence provides preliminary support for the role of SVT in enhancing visual memory and attentional regulation, although these effects are not uniform across attentional components or task types. Compared with sustained attention, SVT may be more effective in improving cognitive processes related to rapid information extraction, short-term retention, and target tracking. Nevertheless, its ecological transfer to real sport settings remains to be further clarified.

### Motor level: enhancement of perception–action coupling efficiency

#### Improvement of reaction speed

SVT may enhance reaction capability by accelerating neural information processing within the perception–decision–execution chain. Zwierko et al. (2024a) conducted a 5-week SVT intervention with 50 elite volleyball players and found that the reaction index (REAC-INDEX) was significantly reduced, with the magnitude of improvement positively correlated with saccadic acceleration (r = 0.41, *p* = 0.007). This suggests that enhanced visual search ability may represent a key mechanism underlying faster reaction performance (Zwierko et al., 2024a). However, another study by the same group reported that this effect disappeared 4 weeks after the intervention and was unrelated to lower-limb explosive power (Zwierko et al., 2024b), indicating that the observed gains may mainly reflect short-term neural adaptations rather than broader improvements in physical capacity.

In contrast, Hülsdünker, Gunasekara & Mierau (2021a) conducted a 19-week intervention in adolescent badminton players and found that improvements in reaction time persisted for more than 6 weeks. Electromyography analysis further showed that this improvement primarily resulted from a shortening of the pre-motor phase rather than changes in the motor phase (Hülsdünker, Gunasekara & Mierau, 2021a). This finding supports the view that SVT may promote more stable neural adaptation by enhancing information transmission efficiency within the central visuomotor network.

It is also noteworthy that the effects of SVT on reaction performance may be directly influenced by training parameters. One study compared visuomotor reaction times in 22 healthy individuals under normal vision and three different stroboscopic conditions across three separate testing days. The results showed that reaction time became slower as stroboscopic difficulty increased, indicating that stroboscopic parameters can directly influence visuomotor performance and suggesting a possible dose–response relationship between SVT difficulty and task performance (Büchel, Hülsdünker & Baumeister, 2025). This finding may partly explain the inconsistency across studies, as the effects of SVT depend not only on whether training is applied, but also on stroboscopic intensity, task demands, and individual adaptation level.

#### Improvement of hand–eye coordination ability

Eye–hand coordination (EHC) reflects the coordinated interaction between the visual and motor systems and is essential for executing complex motor skills and responding efficiently to external stimuli (Sun, Wang & Zhao, 2018). Current evidence suggests that SVT has some potential to improve EHC, although its effects appear to be task- and training-specific.

Appelbaum et al. (2016) conducted an 11-week SVT program and found significant improvements in dynamic visual acuity and inhibitory control in softball players, but no significant change in a standardized EHC clicking task. This suggests that the benefits induced by SVT may not readily transfer to simplified two-dimensional laboratory coordination tasks. By contrast, Ellison et al. (2020) found that a single session of stroboscopic intervention significantly improved EHC performance in ice hockey players (*p* < 0.05), and that this effect persisted for up to 10 days. However, the improvement did not transfer to an untrained visual search task, again indicating that the benefits of SVT for EHC may be largely confined to tasks sharing similar perception–action characteristics.

Mechanistically, such gains may arise because athletes learn to use temporal information and key visual cues more efficiently under intermittent visual conditions. By optimizing temporal anticipation and action timing, SVT may strengthen perception–action coupling and thereby improve EHC performance. Notably, participants with higher baseline EHC showed greater training gains, suggesting that individual differences in baseline ability and neuroplasticity may be important moderators of SVT responsiveness (Ellison et al., 2020).

Overall, current evidence indicates that SVT has the potential to improve sport-related abilities such as reaction speed and eye–hand coordination, but its effects are not universally consistent and show clear task boundaries and inter-individual variability. First, the benefits of SVT are highly task-specific and are more likely to emerge in tasks closely matched to the training content than in generalized laboratory measures. Second, the durability of training effects varies, with some benefits reflecting short-term neural modulation and more stable adaptation likely requiring longer intervention periods. Third, factors such as stroboscopic parameters, task demands, and baseline ability may all influence the magnitude of training responses. Therefore, while SVT appears to be a promising perception–action training tool, its practical effectiveness remains highly dependent on training context, intervention design, and individual characteristics.

Taken together, the evidence for SVT-related cognitive effects appears to be strongest for task-specific anticipation, selected visual information-processing outcomes, and visuomotor reaction measures. In contrast, findings for broader attentional control, multiple-object tracking, generalized decision-making, and far-transfer outcomes are less consistent. These inconsistencies may partly reflect differences in study design, participant expertise, SVT dosage, task similarity between training and testing, and the presence or absence of follow-up assessment. Therefore, improvements observed in laboratory or sport-specific perceptual-cognitive tasks should not be interpreted as direct evidence of broad cognitive transfer.

## Effects of svt on sport performance

A growing body of empirical evidence suggests that SVT may influence sport-related performance outcomes under specific conditions (see Table 2). Its core rationale is that repeated performance under restricted visual input may induce adaptive changes in visual processing and perception–action coordination, thereby facilitating performance under normal viewing conditions. However, these effects are clearly sport- and task-specific, and their transfer to complex competitive settings remains uncertain.

In soccer, the effectiveness of SVT appears to vary according to player role, task context, and training format. Evidence from goalkeeper-focused studies is generally positive. Elhachemi & Issam (2024) reported that a 1-week SVT intervention in U13 goalkeepers improved motor reaction time and ball-control accuracy, with ball-control performance increasing by 45.7%. Similarly, Wilkins, Nelson & Tweddle (2018) found that, after a 7-week SVT program in six elite goalkeepers, reaction time decreased by 0.044 s in the stroboscopic group but increased by 0.014 s in the control group, suggesting a potential benefit for rapid-response regulation in high-tempo situations.

By contrast, findings in outfield players and soccer-specific field tasks are less consistent. Acute restricted-vision studies further illustrate this task-dependent pattern. Fransen et al. (2017) reported impaired dribbling performance in youth soccer players, particularly among faster dribblers, whereas Beavan et al. (2021) found that stroboscopic vision impaired football-specific passing performance, especially in higher-skilled players. A 4-week stroboscopic training program also did not improve dribbling time or ball-contact frequency in youth soccer players (Palmer, Coutts & Fransen, 2022). However, Fortes et al. (2023) reported that, following an 8-week structured program in professional players, the stroboscopic group showed greater improvements than the control group in sport-specific anticipation tasks, with eye-tracking data showing fewer fixations on opponents, which may indicate more efficient attentional allocation. Zwierko et al. (2024c) further showed that a brief warm-up SVT protocol improved reaction agility under ball-control conditions, including under fatigue, but had no significant effect on off-ball tasks or Illinois agility test performance. Together, these findings indicate that the effects of SVT in soccer are strongly moderated by player role, task demands, visual-restriction format, training context, and intervention duration.

Recent studies have also explored combined-training approaches. Jothi, Dhakshinamoorthy & Kothandaraman (2025b) found that combining SVT with proprioceptive training over 8 weeks significantly improved functional balance, agility, and single-leg hopping ability, with greater benefits than single-modality training. Similarly, Lu et al. (2025) reported that SVT combined with balance training over 12 weeks significantly improved multiple balance-related outcomes, including the Stork Stand Balance Test, Static Single Stance Test, Y-Balance Test, Sensory Organization Test, and Adaptation Test. These findings suggest that SVT may act as a neural catalyst, enhancing reliance on proprioceptive and vestibular inputs and amplifying the effects of complementary training.

Support for this combined-training perspective also comes from other team sports. In basketball, integrating SVT with basketball-specific training for 8 weeks reduced coordination test time and error rate, while also improving 505 change-of-direction performance and decision-making in 3D tactical animation tasks, compared with sport-specific training alone and conventional training (Li et al., 2026). This suggests that embedding SVT within sport-specific practice may enhance perceptual-motor performance and reinforce perception–action coupling in ecologically relevant contexts. Consistent with this task-specific interpretation, Dingirdan Gultekinler, Uzlasir & Tunay (2026) reported that integrating stroboscopic visual perturbation into a basketball-specific neuromuscular warm-up selectively improved the temporal components of reactive agility, including reaction time and decision-making time, but did not provide additional benefits for overall cognitive performance, jump performance, change-of-direction ability, or postural stability. At the same time, the variability across studies indicates that the effectiveness of SVT likely depends on the skill structure, task constraints, and movement characteristics of the sport.

In baseball and softball, several studies have reported positive effects of SVT on sport-specific performance. Clark et al. (2012) found that SVT combined with other visual training methods improved players’ on-base percentage. Edgerton (2018) reported that a 4-week training program improved performance by 20.3% in expert softball players and by 23.7% in novices, suggesting that SVT may be adaptable across skill levels. Other studies using 7–25 progressive sessions combined with sport-specific assessments found improvements in pitch angle and hitting distance in baseball players, with some gains persisting into subsequent competitive seasons (Liu et al., 2020). More recently, Dendy et al. (2025) reported in a longitudinal single-case study that a 16-year-old softball player who completed SVT 2 to 3 times per week throughout an entire season improved batting average by 136% and slugging percentage by 168%, along with gains in ocular convergence range. Although the level of evidence remains limited, these findings support the potential of SVT to improve sport-specific batting performance.

In other sports, the evidence is more mixed but still suggestive. In ice hockey, Mitroff et al. (2013) observed improved shooting accuracy following SVT. In curling, Li et al. (2024) reported that 4 weeks of SVT improved temporal judgment and stone-release control, although throwing accuracy remained inferior to that achieved through temporal feedback training. In volleyball, Kroll et al. (2023) found that 4 Hz SVT during depth-jump training reduced explosive performance while increasing ground reaction force and contact time, suggesting that visual restriction may alter neuromuscular control strategies during strength-related tasks. A series of studies by Zwierko et al. (2024a, 2024b) further showed that 6 weeks of SVT improved simple reaction time, complex reaction time, saccadic dynamics, reaction agility, and sport-specific blocking performance in volleyball players. In addition, several pre–post studies reported that a 12-week SVT intervention improved visual reaction time, dynamic visual acuity, peripheral vision, and depth-perception accuracy in elite volleyball players (Azzem et al., 2026). In sports such as tennis, badminton, and rock climbing, SVT has also been shown to improve perceptual abilities or shorten visuomotor reaction time, although direct evidence for improvements in competition performance remains limited (Vasile & Stănescu, 2024; Schootemeijer & Visch, 2017).

Overall, existing evidence suggests that SVT can enhance sport-related perceptual-cognitive and motor abilities, particularly in training and controlled performance settings. However, its transfer to real competitive environments remains insufficiently established (Fransen, 2024). Differences in ecological validity, task openness, sport-specific skill demands, and individual characteristics likely define the boundary conditions of SVT effectiveness.

The evidence for sport-performance transfer remains heterogeneous. Controlled longitudinal studies with sport-specific outcomes provide stronger support for possible task-specific benefits of SVT, particularly when training and testing share similar perceptual and motor demands. In contrast, acute visual-restriction studies, case reports, multi-component interventions, and studies without follow-up mainly inform task difficulty, feasibility, or hypothesis generation rather than established performance enhancement. Accordingly, sport-specific task improvement should not be equated with meaningful improvement in real competitive performance.

## Neurophysiological mechanisms of svt

Although numerous studies have reported positive effects of SVT on cognitive and motor performance, its underlying neurophysiological mechanisms remain insufficiently understood. At present, electroencephalography (EEG)-based studies provide the main evidence for explaining how SVT influences neural processing. By examining changes in the latency and amplitude of specific event-related potential (ERP) components, researchers can assess indirect neurophysiological indices related to visual processing speed, neural resource allocation, and possible network-level adaptation. A conceptual overview of the potential mechanisms is shown in Fig. 2.

**Figure 2:**
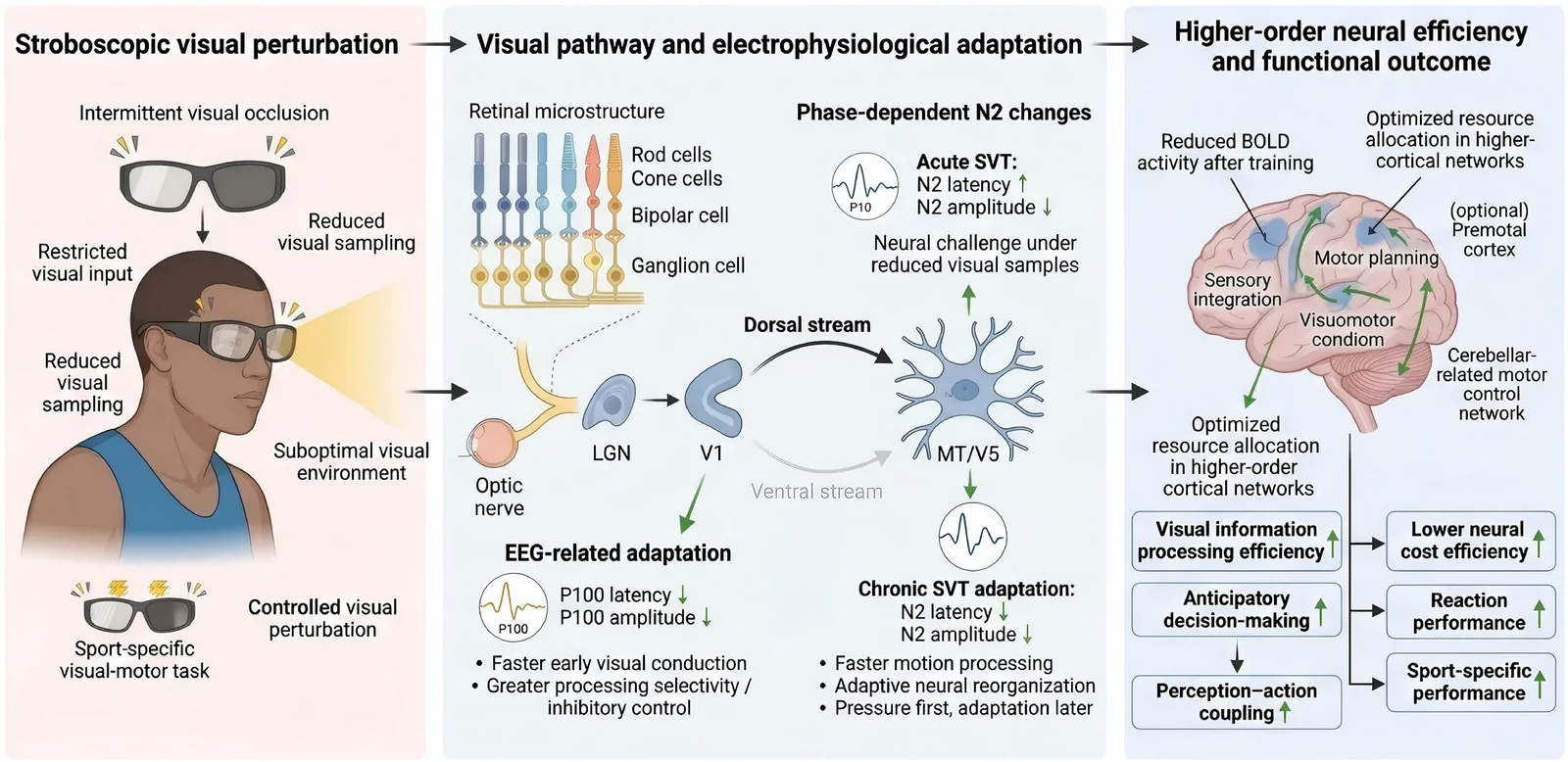
Potential neurophysiological mechanisms of stroboscopic visual training. ↑ indicates an increase; ↓ indicates a decrease. LGN, lateral geniculate nucleus; V1, primary visual cortex; MT/V5, middle temporal visual area; EEG, electroencephalography; ERP, event-related potential; fMRI, functional magnetic resonance imaging; BOLD, blood-oxygen-level-dependent signal. N2 latency refers to the time from visual stimulus onset to the peak of the N2 component, whereas N2 amplitude refers to the magnitude of the negative voltage deflection relative to baseline. P100 latency refers to the time from visual stimulus onset to the P100 peak, whereas P100 amplitude refers to the voltage change of the P100 peak relative to baseline.

From a neuroanatomical perspective, visual information is initially received by retinal photoreceptors and converted into electrical signals. These signals are then relayed through the lateral geniculate nucleus (LGN) and projected to the primary visual cortex (V1) for early-stage visual analysis (Hall & Hall, 2020; Kandel, Schwartz & Jessell, 2000). Processed information is subsequently transmitted along the dorsal visual pathway to the middle temporal visual area (MT/V5), which plays a central role in higher-order motion processing (Hebart & Hesselmann, 2012). Accordingly, current mechanistic research has mainly focused on two key regions of the visual pathway: V1, which is associated with early visual processing, and MT/V5, which is involved in motion-sensitive processing.

### Middle temporal visual area: changes in N2 latency and amplitude

The MT/V5 region is a core cortical area for processing visual motion information, and its activity can be indexed by the N2 component of motion-evoked potentials. N2 latency reflects the speed of motion processing, whereas N2 amplitude reflects the magnitude of neural activation. Existing evidence suggests that SVT can alter both indices.

The available evidence suggests a possible pattern of initial perceptual stress followed by training-related changes, although this interpretation is based on a limited number of studies. In an acute intervention study, Hülsdünker, Fontaine & Mierau (2023) compared EEG responses under stroboscopic vision, screen-shutter vision, and natural vision in 26 university students. Under the stroboscopic condition, reaction time increased significantly, N2 latency was prolonged, and N2 amplitude was reduced. The authors suggested that intermittent occlusion reduced available visual information, forcing the brain to rely on fewer visual samples and thus increasing immediate processing demands (Hülsdünker, Fontaine & Mierau, 2023). Interestingly, cortical EEG analysis further showed that significant V1 activation was observed mainly under baseline and shutter conditions, whereas the stroboscopic condition elicited relatively greater activation in MT/V5. This finding suggests that SVT may preferentially engage visuomotor circuitry and modulate temporal encoding in motion-sensitive regions (Hülsdünker, Fontaine & Mierau, 2023).

After repeated exposure, however, the nervous system appears to adapt to intermittent visual input. In a 10-week intervention study with a 6-week retention period, Hülsdünker, Gunasekara & Mierau (2021b) found that SVT significantly shortened N2 and N2-r latencies, while no significant changes were observed in motor cortex activation. This indicates that the improvement in reaction speed may primarily result from faster visual information processing rather than direct enhancement of motor execution. Notably, reaction time improvements were strongly associated with reductions in N2/N2-r latency, providing convergent support for a link between behavioral gains and changes in neural processing (Hülsdünker, Gunasekara & Mierau, 2021b). Nevertheless, the relatively small sample size (*N* = 32) limits the stability of this conclusion and highlights the need for larger replication studies.

Overall, current evidence suggests that SVT may be associated with changes in MT/V5-related motion processing. Acute exposure may increase perceptual demands and prolong N2 latency, whereas repeated training has been associated with shorter N2/N2-r latency and faster visuomotor responses. However, these findings should be interpreted as preliminary because they are based on limited samples and indirect ERP indices. Reductions in N2 amplitude may reflect altered neural resource allocation, but they do not by themselves demonstrate greater neural selectivity or adaptive reorganization.

### Primary visual cortex: dynamic modulation of P100 latency and amplitude

The P100 component of visual evoked potentials is a classical marker of neural conduction speed and processing efficiency in the primary visual cortex (V1). Shorter P100 latency is generally interpreted as evidence of faster neural transmission and enhanced early visual processing (Baiano & Zeppieri, 2022).

Poltavski, Biberdorf & Praus Poltavski (2021) reported that a 10-week stroboscopic intervention in young ice hockey players significantly reduced both P100 latency and amplitude, suggesting that SVT can modulate early visual pathway function. Similarly, Zwierko et al. (2023) conducted a 6-week SVT program in 22 handball athletes and found that P100 latency in the dominant eye decreased from 105.6 to 103.9 ms. Under binocular visual field conditions, latency shortening was also observed in the parafoveal region (103.7 to 102.8 ms), whereas no significant changes were found in the non-dominant eye (Zwierko et al., 2023). These results suggest that SVT may preferentially affect visual pathways with stronger structural or functional advantages.

Because P100 latency reflects neural conduction from the retina to the visual cortex, the observed shortening may indicate faster signal transmission and more efficient early-stage visual processing. One possible explanation is that intermittent visual occlusion forces the visual system to extract and integrate information within brief time windows, which may contribute to changes in V1-related processing. The preferential effect observed in the dominant eye may be related to pre-existing structural and functional advantages, such as greater efficiency in retinal–cortical transmission (Liu et al., 2021). In contrast, the reduction in P100 amplitude may reflect altered allocation of visual processing resources, potentially involving increased selectivity or inhibitory control during visual processing. This interpretation is consistent with the “faster but weaker” P100 pattern reported by both Zwierko et al. (2023) and Poltavski, Biberdorf & Praus Poltavski (2021). However, these interpretations remain indirect, and alternative explanations such as attention, task familiarity, or measurement variability cannot be excluded. Therefore, current V1-related evidence should be viewed as preliminary support for possible changes in early visual processing rather than direct proof of neuroplastic adaptation.

### Summary and limitations of current neurophysiological evidence

Taken together, current neurophysiological evidence suggests that SVT may be associated with changes in early and motion-related visual processing. Shorter N2/N2-r and P100 latencies may reflect faster visual processing, whereas amplitude changes may indicate altered neural resource allocation (Hülsdünker, Gunasekara & Mierau, 2021b; Zwierko et al., 2023; Poltavski, Biberdorf & Praus Poltavski, 2021). However, ERP indices provide indirect evidence and do not by themselves prove neural efficiency or adaptive reorganization.

The current evidence base also remains limited in depth and scope. Most mechanistic studies rely on EEG, which offers excellent temporal resolution but limited spatial precision. A small number of studies have begun to apply fMRI. For example, Wohl et al. (2024) reported reduced blood-oxygen-level-dependent signal (BOLD) responses in regions associated with sensory integration and motor planning after visual perturbation neuromuscular training. This finding may be consistent with improved neural efficiency, but alternative explanations such as task familiarity, altered strategy, reduced effort, or speed–accuracy trade-offs cannot be excluded.

Although this finding offers a promising new direction for mechanistic research, its generalizability remains limited by the small sample size (*N* = 8) and the relatively simplified task context. Therefore, future studies should combine EEG, fMRI, and other multimodal neuroimaging approaches to clarify how SVT may influence visual and visuomotor networks across different populations, task demands, and training durations.

## Key issues to be addressed in the application of svt

Although existing studies have demonstrated the potential of SVT to enhance cognitive function and sport performance, several important issues remain unresolved. Addressing these challenges is essential for optimizing intervention design and advancing the evidence base for SVT.

### Lack of clear standards for training parameters

Considerable variability exists in the stroboscopic parameters used across SVT studies, including occlusion frequency, duty cycle, session duration, and overall intervention length. To date, no clear design framework or dose–response model has been established. Reported frequencies range from 0.2 to 30 Hz, and interventions vary from a single 5-min session to programs lasting more than 19 weeks. As a result, key practical questions remain unanswered, including the optimal occlusion frequency, session duration, and training period. Differences in occlusion settings, task type, and stimulus synchronization may also partly explain the inconsistency across findings. Moreover, evidence suggests that greater stroboscopic difficulty increases task cost and may reduce reliability under highly demanding conditions (Büchel, Hülsdünker & Baumeister, 2025). Future research should therefore move beyond fixed, one-size-fits-all protocols and adopt progressive, individualized parameter adjustment based on performance thresholds. Establishing a dose model that integrates frequency, effect, and population characteristics may help define the optimal boundaries and combinations of SVT prescription across different groups and task contexts.

### Task transfer mechanisms remain unclear

The effects of SVT appear to be highly task-specific. In many studies, performance gains are evident only in trained tasks, whereas transfer to untrained contexts is limited. This substantially restricts the practical value of SVT, particularly in competitive settings where broad transfer is often the ultimate goal. Future research should therefore focus more explicitly on the mechanisms and boundaries of transfer, including the moderating roles of cognitive demands, task characteristics, and intervention structure. Multi-task training paradigms may provide one possible strategy for enhancing cross-task generalization.

### Durability of training effects has been insufficiently examined

Most existing studies have focused on the immediate or short-term effects of SVT, while relatively few have included delayed post-tests or long-term follow-up. Available evidence suggests that some training benefits may decline within days or weeks after the intervention, especially when training duration is short or stimulus intensity is insufficient to induce stable neural adaptation. Future studies should therefore include delayed assessments at multiple time points to map the decay trajectory of SVT-related gains and to identify the mechanisms underlying their retention. In addition, maintenance strategies and the role of individual characteristics in the persistence of training effects should be examined in order to develop more sustainable and individualized intervention models.

### Neurophysiological mechanisms require further clarification

Current mechanistic research on SVT remains largely limited to single-modality methods. A more informative approach would involve multimodal integration, particularly combining the high temporal resolution of EEG, which helps explain when neural responses occur, with the high spatial resolution of fMRI, which can clarify where neural changes take place. Such an approach may help clarify the pathway linking SVT intervention, neural processing changes, and behavioral outcomes. Advancing this line of research will be essential for opening the neurophysiological “black box” of SVT and for supporting its scientific application in sport.

### Methodological limitations and potential publication bias

As summarized in Tables S1 and S2, the current SVT literature remains methodologically heterogeneous. Common limitations include small samples, short intervention periods, incomplete reporting of stroboscopic parameters, limited follow-up, and insufficient use of active control groups or blinded outcome assessment. In addition, because SVT is an emerging and attractive training approach, positive findings may be more likely to be published than null or negative findings. This potential publication bias may lead to an overestimation of SVT effectiveness. Future studies should therefore use preregistered protocols, active control conditions, standardized SVT parameter reporting, ecologically valid sport-performance outcomes, and long-term follow-up assessments.

## Conclusion

Based on the current body of empirical evidence, this structured narrative review summarized the effects, application contexts, and potential neurophysiological mechanisms of SVT. The main conclusions are as follows:

(1) In terms of visual processing mechanisms, current evidence suggests that SVT may influence early visual processing and selected perceptual-cognitive functions, including visual short-term memory, attentional allocation, anticipation, and decision-making, particularly in tasks requiring rapid perception–action coupling.
(2) In terms of sport performance, SVT has shown positive effects across several sports that depend on rapid perception and precise judgment, especially in sport-specific training involving high-speed dynamic information. However, its effects remain clearly constrained by task specificity and limited transferability.
(3) In terms of neurophysiological mechanisms, preliminary evidence suggests that SVT may be associated with changes in N2 latency in MT/V5-related motion processing and P100 latency in V1-related early visual processing. Current fMRI evidence remains limited and should be interpreted as exploratory rather than definitive evidence of neural efficiency or adaptive reorganization.

These conclusions should be interpreted cautiously because the current evidence remains heterogeneous, this review was narrative and not preregistered, potential selection or publication bias cannot be excluded, and mechanistic evidence still comes from a limited evidence base. Overall, SVT should be regarded as a promising but still developing perceptual-training approach that can be embedded within sport-specific practice. However, evidence for long-term retention, broad transfer, ecological transfer, and real competitive performance enhancement remains limited. Future studies should use standardized parameter reporting, active control conditions, ecologically valid sport-performance outcomes, and long-term follow-up designs to clarify dose–response relationships and task-transfer mechanisms.

## Supplemental Information

10.7717/peerj.21740/supp-1 Supplemental Information 1 Methodological characteristics and appraisal considerations of included SVT studies on cognitive function. methodological characteristics and appraisal considerations used to guide narrative interpretation. It was not intended as a formal risk-of-bias assessment or quantitative evidence grading. Studies with controlled designs, sport-specific tasks, clearly reported SVT parameters, and follow-up assessments were interpreted more strongly than uncontrolled, acute, case-based, or laboratory-only studies.

10.7717/peerj.21740/supp-2 Supplemental Information 2 Methodological characteristics and appraisal considerations of included SVT studies on sport performance. methodological characteristics and appraisal considerations used to guide narrative interpretation. It was not intended as a formal risk-of-bias assessment or quantitative evidence grading. Studies with controlled designs, sport-specific outcomes, clearly reported SVT parameters, and follow-up assessments were interpreted more strongly than uncontrolled, acute, case-based, multi-component, non-traditional SVT, or laboratory-only studies.

## Additional Information and Declarations

### Data Availability

The following information was supplied regarding data availability:

This is a Literature Review.
