# Supplementary material for: Stroboscopic visual training in sport: a structured narrative review of effects, applications, and potential neurophysiological mechanisms
Source: PeerJ. 2026 Sep 29;14:e21740. doi: 10.7717/peerj.21740 (PMC13632680; doi:10.7717/peerj.21740)
Supplement: Supplementary file 1 [file peerj-14-21740-s001.docx]

| **Study** | **Design** | **Sample size** | **Control condition** | **SVT parameters reported** | **Sport-specific outcome** | **Follow-up** | **Main methodological considerations** |
| --- | --- | --- | --- | --- | --- | --- | --- |
| Vasile & Stanescu (2024) | Non-randomized controlled longitudinal intervention in elite youth climbers | 17 elite youth climbers; 9 experimental, 8 control | Control group continued normal climbing training without additional strobe sessions | Partial/unclear: 20 strobe sessions across 1 year were reported, but frequency, duty cycle, and exact occlusion parameters were insufficiently specified | Yes; on-sight and red-point climbing performance, IRCRA climbing-specific tests, cognitive and motor-cognitive tests | No separate delayed follow-up beyond post-intervention testing | Ecologically relevant elite climbing context and long intervention period. However, the sample was very small, allocation was not clearly randomized, the experimental group received an additional weekly strobe session, and incomplete reporting of strobe settings limits reproducibility. |
| Zwierko et al. (2024a) | Randomized controlled longitudinal intervention with neurophysiological assessment | 22 elite handball players | Control group performed the same handball-specific in-situ tasks under normal vision | Adequate: Senaptec Strobe; progressive frequency/duty-cycle settings across 6 weeks | Partial; training was sport-specific, but the main outcome was VEP/P100 rather than sport performance | Four-week retention assessment | Stronger features include randomization, sport-specific training tasks, VEP measurement, and retention testing. Limitations include small sample size, limited generalizability, and absence of direct competitive-performance outcomes. |
| Zwierko et al. (2024b) | Randomized controlled in-situ intervention focusing on agility-related visuomotor reaction speed | 50 youth volleyball players; 26 males, 24 females | Control group performed volleyball-specific training under normal visual conditions | Adequate: 6-week intervention with progressive stroboscopic settings | Yes/partial; CODS, RA, REAC-INDEX, and saccadic dynamics related to volleyball agility demands | No long-term follow-up clearly reported | Comparatively strong design with randomization, active sport-specific control, field-based agility measures, and adequate sample size for this literature. Limitations include youth-only sample, no blinding or sham eyewear, short intervention period, and possible overlap with related Zwierko volleyball publications. |
| Fortes et al. (2023) | Experimental randomized parallel-group intervention | 28 male professional soccer players | Control group performed comparable soccer training without stroboscopic eyewear | Adequate: Nike Vapor Strobe; 100 ms visible / 210 ms opaque; 8 weeks, 3 sessions per week | Partial; soccer-related perceptual-cognitive tasks were used, but real match performance was not directly assessed | No delayed follow-up reported | Randomized design and professional soccer sample strengthen relevance. However, no delayed retention test, no neurophysiological measures, possible learning effects, and limited ecological transfer because outcomes were perceptual-cognitive tasks rather than real-game behavior. |
| Hulsdunker et al. (2023) | Acute within-subject experimental study with EEG-based mechanistic assessment | 26 participants | Baseline normal vision and screen-shutter condition controlled for visual motion information independent of eyewear | Complete: 5 Hz stroboscopic condition with 40% duty cycle; EEG acquisition reported | No; laboratory visuomotor reaction task | No longitudinal follow-up | Strong mechanistic design with within-subject control and direct EEG measurement of N2 latency/amplitude. However, this was an acute laboratory study rather than a training intervention, and findings should not be interpreted as direct evidence of long-term neural adaptation. |
| Zwierko et al. (2023) | Randomized controlled in-situ intervention | 50 youth volleyball players | Control group performed the same volleyball-specific training under normal vision | Complete: Senaptec Strobe; progressive frequency and duty-cycle settings across 6 weeks | Partial/yes; included laboratory visual/visuomotor tests and field reactive-agility testing | Four-week retention assessment | Randomization, active control, sport-specific training, field outcome, and retention test strengthen the study. Limitations include youth-only sample, absence of placebo/sham eyewear or blinding, no expectancy assessment, and uncertain generalizability to adult elite athletes. |
| Hulsdunker et al. (2021a) | Controlled longitudinal intervention; Part 1 focused on reaction and behavior | 45 elite youth badminton athletes participated; 32 included in final analysis | Control group completed identical badminton-specific training under normal vision | Adequate: dynamically adjusted from approximately 15 Hz to 8 Hz; 10 weeks, average 45 min/week | Partial/yes; laboratory VMRT and EMG outcomes plus badminton-specific field tests | Six-week retention interval | Elite sample, active sport-specific control, 10-week intervention, and retention assessment strengthen the study. However, final sample was modest after attrition, field-test improvements were not clearly different between groups, and absence of blinding may introduce expectancy effects. |
| Hulsdunker et al. (2021b) | Controlled longitudinal mechanistic study; Part 2 focused on brain-behavior mechanisms | Same cohort as Part 1: 45 participated, 32 included in final analysis | Control group completed identical training under normal vision | Adequate: same 10-week stroboscopic badminton intervention as Part 1 | Partial; neurophysiological and visuomotor reaction tasks, but not direct competition performance | Six-week retention interval | Direct EEG measurement of N2/N2-r and BA6 negativity strengthens mechanistic interpretation. However, the sample overlaps with Part 1 and should not be treated as fully independent evidence. Mechanistic findings are laboratory-based and should be interpreted as indirect support for visual-processing adaptation. |
| Ellison et al. (2020) | Randomized controlled acute intervention | 62 male physically active participants; 31 strobe, 31 control | Control group completed the same EHC task with unimpaired vision | Complete: Nike Vapor Strobe; 100 ms visible / 150 ms opaque; four training sessions with 480 total light responses | Partial; eye-hand coordination task was performance-relevant but not sport-specific | Immediate, 10 min, and 10 days post-training; visual-search transfer test at 10 days | Randomization, adequate sample size, repeated post-tests, and transfer assessment strengthen interpretation. Limitations include male-only participants, acute exposure, practiced-task specificity, and absence of transfer to the untrained visual-search task. |
| Ballester et al. (2017) | Acute within-subject, counterbalanced experimental condition comparison | 20 male undergraduate students | Normal-vision condition served as within-subject comparator | Adequate: Nike Vapor Strobe; 4 Hz condition reported | No; laboratory coincidence-anticipation and sustained-attention task | No post-training retention; only time-on-task within session | Counterbalanced within-subject design controls inter-individual variability. However, this was not a longitudinal training study, involved a small non-athlete sample, used laboratory outcomes only, and does not directly address transfer to normal-vision sport performance. |
| Appelbaum et al. (2016) | Exploratory applied cohort study in collegiate softball | 25 collegiate softball players; 15 SVT, 10 control | Control teammates completed regular softball training without SVT drills | Partial: Nike Vapor Strobe; initial level 3 reported, but individual progression and total exposure varied | Partial; sensorimotor battery was relevant to sport but not direct softball performance | No delayed follow-up reported | Applied team setting improves ecological relevance. However, the study was exploratory, non-randomized, and based on secondary analysis; the sample was small, no active sham control was used, training was multi-component, and attribution specifically to stroboscopic exposure is limited. |
| Wilkins & Gray (2015) | Controlled pre-post intervention comparing two stroboscopic training modes | 30 athletes; 12 females, 18 males; mean age 22.5 years | Constant strobe rate group served as active comparison; no normal-vision or transparent-eyewear control group | Adequate/partial: PLATO occlusion glasses; VSR group progressed across levels, CSR group remained at Level 1; 10 sessions over 6 weeks, 20 min/session | Partial; one-handed catching task was sport-relevant, but UFOV and MIDS were laboratory perceptual-cognitive tests | No delayed follow-up reported | Active comparison between stroboscopic protocols is useful, and motivation/effort were assessed. However, the lack of a normal-vision or sham-control group limits attribution to SVT. No between-group effects were found, and findings rely partly on correlations between perceptual changes and catching improvement. |
| Holliday (2013) | Small controlled pre-post training study; graduate report | 16 participants divided into experimental and control groups | Control group trained without the stroboscopic effect | Unclear/partial: Nike Vapor Strobe used, but detailed strobe settings were not clearly reported | Partial; DVA was laboratory-based, with ball-catching performance also assessed | Immediate after first session, post-program, and two-week post-training assessments | Provides early evidence on DVA and catching performance, but methodological strength is limited by very small sample size, graduate-report status, incomplete reporting of strobe parameters, limited control structure, and possible learning or ball-training effects. |
| Appelbaum et al. (2012) | Controlled experimental study with immediate and delayed memory assessment | Experiment 1 included 84 participants; additional delayed-retention cohort in Experiment 2 | Control group performed identical training with transparent non-stroboscopic eyewear | Complete: Nike Vapor Strobe; 100 ms clear state; opaque state varied across levels | No; laboratory partial-report visual-memory task | 24-h delayed assessment in Experiment 2 | Transparent-eyewear control and delayed assessment strengthen interpretation. However, outcomes were laboratory-based, participants were mixed across community and varsity cohorts, transfer to sport-specific performance was not tested, and some improvements may reflect task practice rather than SVT-specific effects. |
| Smith & Mitroff (2012) | Randomized controlled acute intervention; pre-test, training, and repeated post-tests | 30 university community participants; 15 strobe, 15 control | Control group completed the same anticipatory timing task under normal vision | Complete: Nike Vapor Strobe; 100 ms visible / 150 ms opaque; single 5-7 min exposure | No; laboratory Bassin Anticipation Timer task | Immediate, 10 min, and 10 days post-training | Randomized design and retention testing strengthen interpretation. However, the sample was small and non-athletic, the intervention was acute, the outcome was laboratory-based, and the effect was short-lived, with no clear advantage at 10 days. |
| Appelbaum et al. (2011) | Controlled exploratory multi-cohort training study | 157 participants across laboratory and sport-team cohorts | Control participants completed identical training with transparent non-stroboscopic eyewear | Adequate: Nike Vapor Strobe; levels 1-6; 100 ms transparent state and 67-900 ms opaque state; exposure varied by cohort | No direct sport-performance outcome; laboratory cognitive and perceptual tasks | No delayed follow-up; post-tests were immediate | Large sample and transparent-eyewear control strengthen interpretation. However, the study combined multiple cohorts and training protocols, outcomes were laboratory-based, and improvements were selective rather than general. Benefits were observed for central motion sensitivity and transient attention, but not for peripheral motion sensitivity or sustained attention/MOT, indicating limited transfer. |
|  |  |  |  |  |  |  |  |
