# Supplementary material for: Stroboscopic visual training in sport: a structured narrative review of effects, applications, and potential neurophysiological mechanisms
Source: PeerJ. 2026 Sep 29;14:e21740. doi: 10.7717/peerj.21740 (PMC13632680; doi:10.7717/peerj.21740)
Supplement: Supplementary file 2 [file peerj-14-21740-s002.docx]

| **Study** | **Design** | **Sample size** | **Control condition** | **SVT parameters reported** | **Sport-specific outcome** | **Follow-up** | **Main methodological considerations** |
| --- | --- | --- | --- | --- | --- | --- | --- |
| Dendy et al. (2025) | Single-subject case study across one softball season | 1 female varsity softball player, aged 16 years | Historical comparison with previous season; no concurrent control | Partial/unclear: stroboscopic goggles worn 2-3 times/week during hitting and fielding, but exact frequency, duty cycle, and total exposure were not fully specified | Yes; batting average, on-base percentage, slugging percentage, hard-hit ball percentage, and vision-specific measures | Season-level comparison, but no controlled follow-up | Highly ecological on-field case evidence, but very low methodological strength because of single-subject design, no randomization, no concurrent control, possible maturation/coaching effects, and inability to isolate SVT from normal seasonal development. |
| Jothi et al. (2025) | Controlled pre-post intervention study | 98 male football players; 49 experimental, 49 control | Control group continued regular football training | Partial: Senaptec Strobe used, but detailed frequency, duty cycle, progression, and total exposure were insufficiently specified | Partial/yes; balance, agility, and single-leg hop tests are performance-related but not direct match performance | No delayed follow-up | Large sample strengthens the evidence. However, the experimental intervention combined SVT with proprioceptive training, so the independent effect of SVT cannot be isolated. Lack of detailed SVT parameter reporting and absence of follow-up reduce interpretability. |
| Rodrigues et al. (2025) | Randomized controlled 6-week intervention using light-based visual-processing manipulation | 24 professional soccer players | Control group performed same soccer training under normal lighting | Not conventional SVT: Okkulo system used specific lighting to slow visual processing rather than intermittent stroboscopic eyewear | Partial; visual and visuomotor outcomes, not direct on-field soccer performance | No delayed follow-up | Randomized design and professional soccer context are strengths. However, this is not traditional eyewear-based SVT, and transfer to on-field or match performance was not tested. Mixed sex/team contexts and modest sample size increase heterogeneity. |
| Elhachemi & Issam (2024) | Pre-post experimental intervention in youth soccer goalkeepers | U13 soccer goalkeepers; exact sample allocation requires confirmation from full text | No clearly described independent control group in abstracted information | Partial/unclear: Senaptec/stroboscopic glasses used, but frequency, duty cycle, total exposure, and progression were not clearly reported | Yes; goalkeeper reaction speed and ball-control/holding skill | No follow-up reported | Provides sport-specific youth goalkeeper evidence, but methodological transparency is limited. The study appears to rely mainly on pre-post comparisons, lacks clear randomization/control details, and insufficiently reports SVT parameters. Results should be interpreted cautiously. |
| Harrison et al. (2024) | Acute within-subject biomechanical experiment | 20 young adults; 11 males, 9 females | Full-vision condition served as within-subject comparator | Adequate: 3 Hz stroboscopic vision during depth jumps | Partial; depth jump biomechanics are performance- and rehabilitation-relevant, but not competition outcomes | No follow-up; acute exposure only | Objective GRF/EMG measures strengthen internal validity. However, this was not a training study, used a general young-adult sample, and focused on acute sensorimotor disruption rather than performance enhancement or long-term adaptation. |
| Li et al. (2024, Life) | Cross-over controlled acute experiment under stroboscopic and normal visual conditions | 32 national-level male curling athletes | Each participant completed both normal-vision and stroboscopic conditions | Partial/adequate: stroboscopic condition applied, but settings were mainly described as condition rather than detailed training dosage | Yes; time perception error, delivery speed-control error, and curling accuracy | No follow-up; acute condition only | Strong sport-specific relevance and cross-over design. However, this was an acute visual-perturbation study rather than a training intervention. Stroboscopic vision worsened time perception, speed control, and accuracy, so findings mainly indicate task difficulty and feasibility for training design. |
| Li et al. (2024, Scientific Reports) | Randomized controlled 4-week intervention with three groups | 30 national-level curling athletes | Temporal feedback training group and control group | Partial/adequate: stroboscopic glasses used; levels increased weekly from Level 1 to Level 4; exact frequency/duty cycle not fully specified | Yes; duration judgment, delivery speed control, and curling delivery accuracy | No delayed follow-up | Strong sport-specific relevance and inclusion of active comparison condition are strengths. However, sample size was modest, strobe settings were described by levels rather than exact parameters, and SVT improved duration judgment/speed control more clearly than delivery accuracy. |
| Wohl et al. (2024) | Controlled laboratory pilot intervention combining visual perturbation neuromuscular training and fMRI assessment | 8 healthy female recreational athletes with high-risk landing mechanics | No independent control group; pre-post design | Partial: VPNT used stroboscopic glasses plus external visual-focus feedback across four sessions | Partial/yes; LESS landing mechanics are injury-risk and performance-relevant, but not direct sport competition outcomes | No long-term follow-up | Innovative integration of landing mechanics and fMRI provides useful mechanistic insight. However, sample was very small, no randomized control group was included, and the intervention combined stroboscopic glasses with external-focus neuromuscular feedback, limiting attribution specifically to SVT. |
| Zwierko et al. (2024, soccer warm-up) | Acute randomized/counterbalanced experimental study during soccer warm-up | 22 male soccer players | Standard warm-up under normal vision compared with stroboscopic warm-up | Partial: stroboscopic eyewear during ball-specific warm-up; exact frequency/duty-cycle should be checked in full method | Yes; reactive agility with/without ball and fatigued/non-fatigued conditions | No follow-up; acute warm-up effect only | Strong task-specific soccer agility design. However, effects were acute and task-specific, mainly for reactive agility with dribbling. No clear benefit was observed for planned agility or non-ball tasks; long-term transfer remains unknown. |
| Zwierko et al. (2024, volleyball blocking) | Randomized controlled 6-week intervention on volleyball-specific blocking reaction speed | 50 young volleyball players; 26 males, 24 females | Control group performed same volleyball-specific training under normal visual conditions | Adequate: 6-week stroboscopic program; parameters sufficiently described for intervention interpretation | Yes; volleyball-specific blocking reaction speed; CMJA assessed as possible motor contributor | Four-week retention test | Active control, sport-specific task design, and retention testing strengthen interpretation. However, effects appeared short-term and were not clearly maintained at retention. Youth-only sample, no sham eyewear/blinding, and possible overlap with related Zwierko volleyball studies should be noted. |
| Kroll et al. (2023; first online 2020) | Acute within-subject repeated-measures biomechanical study | 13 female NCAA Division I volleyball athletes | Full-vision condition compared with stroboscopic conditions | Adequate: full vision, 4 Hz, and 1.75 Hz stroboscopic conditions | Partial; RSI, jump height, contact time, GRF, and RFD are sport-relevant but not direct volleyball performance | No follow-up; acute exposure only | NCAA Division I athletes and objective biomechanical outcomes are strengths. However, sample was small and the study examined acute performance under stroboscopic vision rather than training adaptation. Lower RSI/jump height under difficult strobe conditions indicates disruption rather than enhancement. |
| Strainchamps et al. (2023) | Acute within-subject sport-specific warm-up study | 28 international-level table tennis athletes | Each athlete completed normal-vision and stroboscopic-eyewear warm-up conditions | Partial: stroboscopic eyewear applied during 10-min table-tennis-specific warm-up; exact frequency/duty-cycle should be checked | Yes; sport-specific table-tennis reaction-speed test using ball-machine returns | No follow-up; acute warm-up effect only | High-level athlete sample and sport-specific testing are strengths. However, no additional benefit of stroboscopic eyewear was observed beyond normal warm-up, suggesting short-term SVT during warm-up may not produce immediate gains in highly trained athletes. |
| Palmer et al. (2022) | Exploratory randomized controlled 4-week soccer dribbling intervention | 61 youth players initially; 36 included in training study; 18 intervention, 18 control | Control group completed same dribbling training under normal vision | Adequate: Nike Vapor Strobe; baseline testing used Level 3 and Level 7; training progressed from Levels 3-6 across four sessions | Yes; adapted UGent soccer dribbling task, time and number of touches | One-week retention test | Sport-specific outcome and retention test strengthen relevance. However, the study was exploratory and underpowered relative to the a priori target, used a convenience sample, had short intervention duration, and found no beneficial training or retention effect on dribbling performance. |
| Beavan et al. (2021) | Acute within-subject football-specific visual-restriction study | 84 amateur male football players | Normal-vision condition served as within-subject comparator | Partial: stroboscopic condition used, but exact frequency/duty-cycle not evident from abstracted information | Yes; Footbonaut passing accuracy and passing time | No follow-up; acute performance only | Large sample and football-specific testing environment strengthen ecological relevance. However, this was an acute visual-restriction study rather than training. Stroboscopic vision impaired passing accuracy, especially in skilled players, informing task difficulty rather than direct performance improvement. |
| Liu et al. (2020) | Pre-registered randomized placebo-controlled pilot intervention | 24 collegiate baseball players; smaller subsamples for batting practice/game statistics | Placebo drills modeled after prior vision-therapy controls | Partial: dynamic vision training included stroboscopic, anticipatory timing, and eye-quickness drills; SVT-specific exposure cannot be isolated | Yes; batting practice metrics and NCAA game statistics | No long-term post-intervention follow-up beyond season/game outcomes | Strong design features include preregistration, randomization, placebo control, expectation assessment, and sport-specific transfer outcomes. However, intervention was multi-component, so effects cannot be attributed specifically to SVT. Batting practice improved, but no group differences were found in visual-motor battery or NCAA game statistics. |
| Hulsdunker et al. (2019) | Controlled 4-week intervention with behavioral and neurophysiological outcomes | 10 top-level badminton players; 5 intervention, 5 control | Control group performed identical badminton-specific visuomotor training under normal visual conditions | Adequate: shutter-glasses intervention during badminton-specific training tasks | Yes; badminton-specific smash-defense performance; N2 motion-onset VEP assessed | No delayed follow-up | Elite-athlete sport-specific design and VEP assessment strengthen relevance. However, sample size was very small, no delayed retention test was included, and neurophysiological changes did not reach statistical significance. Behavioral effects should be considered promising but preliminary. |
| Bennett et al. (2018) | Two-experiment laboratory study: acute MOT manipulation and perceptual-motor acquisition experiment | Sample size varies by experiment; laboratory participants | Normal vision, Nike Vapor Strobe, PLATO visual occlusion, and intermittent display conditions | Adequate: methods compared 5.6, 3.2, and 1.8 Hz conditions | No direct sport outcome; MOT and multiple-object avoidance were laboratory perceptual-motor tasks | No sport-performance follow-up | Strong mechanistic comparison of visual perturbation versus elimination. However, outcomes were laboratory-based, participants were not sport-specific athletes, and transfer to competitive sport remains uncertain. |
| Edgerton (2018) | Master's thesis; controlled 4-week field-based softball intervention | 12 softball players; 6 stroboscopic, 6 non-stroboscopic; novice/expert subgroups | Non-stroboscopic training group | Adequate/partial: stroboscopic training over 4 weeks, approximately 3 sessions/week; exact settings should be confirmed | Yes; softball ground-ball catching/fielding performance | No delayed follow-up | Field-based softball task and inclusion of skill-level subgroups are useful. However, sample was small, thesis status limits evidence strength, group allocation/blinding were limited, and practice/learning effects may have contributed to improvements. |
| Wilkins et al. (2018) | Pilot case-control study with elite youth football goalkeepers and qualitative interviews | 6 elite youth goalkeepers; 3 SVT, 3 matched controls | Matched control goalkeepers continued normal training | Adequate: Senaptec Strobe; 7-week program, 14 x 45-min sessions plus 1 x 5-min pre-test session; levels 1-8 and eye modes varied | Partial; training involved goalkeeper-specific drills, but measured outcomes were mainly visual/perceptual tests rather than direct save performance | Four-week retention test | Valuable exploratory goalkeeper-specific evidence with retention and qualitative data. However, sample was extremely small, no inferential statistics were used, one participant had rehabilitation-related training modification, and direct sport-performance transfer was not objectively measured. |
| Fransen et al. (2017) | Acute within-subject study of restricted visual feedback during soccer dribbling | 189 youth soccer players aged 10-18 years | Full-vision condition served as within-subject comparator | Complete: Nike Vapor Strobe; Level 3 = 4 Hz, 100 ms clear / 150 ms opaque; Level 7 = 1.33 Hz, 100 ms clear / 650 ms opaque | Yes; Ghent University Dribbling Test | No follow-up; acute condition only | Large sample and sport-specific dribbling task strengthen ecological relevance. However, this was an acute visual-restriction study rather than training. Stroboscopic vision increased dribbling time, especially in faster dribblers, indicating visual-dependence/task-difficulty rather than performance enhancement. |
| Schootemeijer & Visch (2017) | Small controlled sport-specific intervention in skilled youth tennis players | 13 skilled youth tennis players | Control players participated in same training without stroboscopic eyewear | Partial: six stroboscopic training sessions incorporated into regular tennis training; detailed frequency/duty-cycle limited | Yes; forehand/backhand groundstroke hitting accuracy | No follow-up | Direct tennis-specific outcome is a strength. However, sample was very small, study appears to be student/undergraduate research, allocation was complex, and no retention or match-performance outcome was included. |
| Mitroff et al. (2013) | Pilot field study in professional ice hockey players | 11 professional ice hockey players; 6 strobe, 5 control | Control players continued normal training without stroboscopic eyewear | Partial/unclear: Nike SPARQ Vapor Strobe used; eyewear worn at least 10 min/day, but task content/settings were not standardized | Yes; position-specific on-ice shooting or passing accuracy | Post-test approximately 24 h after final exposure; no longer-term follow-up | Highly ecological professional-athlete field study with direct sport-skill outcomes. However, very small sample, no randomization/sham control, possible motivation/placebo effects, and variable training exposure limit causal inference. |
| Clark et al. (2012) | Observational team-level pre-post season comparison using multi-component vision training | University of Cincinnati collegiate baseball team | Historical comparison with previous season and conference-level comparison; no randomized concurrent control | Partial/unclear: strobe glasses were one component of broader vision training including Dynavision, tachistoscope, Brock String, Eyeport, Rotary, near-far training, and saccades | Yes; NCAA batting statistics, including batting average and slugging percentage | Season-level comparison; no controlled follow-up | Real competitive batting statistics provide ecological value. However, intervention was multi-component and not SVT-specific; no randomized control group, no individual SVT dose control, and season-to-season changes may reflect coaching, roster, opponent, or contextual factors. |
